# Multidrug-resistant Tuberculosis in Military Recruits

**DOI:** 10.3201/eid1205.050708

**Published:** 2006-05

**Authors:** Grace Freier, Allen Wright, Gregory Nelson, Eric Brenner, Sundari Mase, Sybil Tasker, Karen L. Matthews, Bruce K. Bohnker

**Affiliations:** * Naval Hospital, Beaufort, South Carolina, USA;; † South Carolina Department of Health and Environmental Control, Columbia, SC, USA;; ‡ California Department of Health, Sacramento, California, USA;; § National Naval Medical Center, Bethesda, Maryland, USA;; ¶ Navy Environmental and Preventive Medicine Unit-2, Norfolk, Virginia, USA

**Keywords:** multidrug-resistant tuberculosis, military medicine, contact investigation, recruit medicine, tuberculosis, chemotherapy, dispatch

## Abstract

We conducted a tuberculosis contact investigation for a female military recruit with an unreported history of multidrug-resistant tuberculosis (MDRTB) and subsequent recurrence. Pertinent issues included identification of likely contacts from separate training phases, uncertainty on latent MDRTB infection treatment regimens and side effects, and subsequent dispersal of the contacts after exposure.

In 2004, a 19-year-old female recruit came to the Naval Hospital in Beaufort, South Carolina, with a history of congestion and rhinorrhea for 4 days. Radiographic examination showed right upper and lower lobe infiltrates. Her initial recruit screening tuberculin skin test (TST) result had been reactive. Consultation with her physician in California indicated similar radiographic findings 2 years earlier; her condition had been diagnosed as smear- and culture-negative tuberculosis (TB). She received oral treatment of 300 mg isoniazid daily, 600 mg rifampin daily, and 1,500 mg pyrazinamide daily for 2 months. After a negative sputum culture, isoniazid and rifampin were continued for 9 months (*1**,**2*). Based on unchanged radiographic findings and 9 months of treatment, her disease was considered to be nonactive and she returned to training. Subsequently, she failed to complete training and was separated from the military.

## The Study

Approximately 3 months after her initial treatment, the index patient was hospitalized in California for TB resistant to isoniazid and rifampin, which met the definition of multidrug-resistant tuberculosis (MDRTB). Initial isolate susceptibility in California showed resistance to isoniazid, rifampin, ethambutol, and streptomycin. Additional isolate susceptibility tests in Denver showed sensitivity to ethionamide, cycloserine, p-amino salicylic acid, clofazimine, levofloxacin, and pyrazinamide, but resistance to isoniazid, rifampin, streptomycin, amikacin/kanamycin, amoxicillin/clavulanate, and rifabutin.

After notification of the recruit's hospitalization in California, Navy personnel began a TB contact investigation (*1*). Recruit populations are highly transient, as persons are frequently added or removed for various medical, dental, legal, or physical performance reasons. Some persons had multiple exposures to the index patient while in the training platoon and subsequently in various processing units. Thus, the contact investigation identified numerous persons who may have had contact with the index patient; these were categorized as "close" or "casual" contacts. Close contacts included persons who shared living quarters with the index patient; casual contacts included persons who had less definable contact with the index patient.

The investigation identified 13 close contact and 8 casual contact new reactors, defined as >5 mm TST indurations in persons who had negative tests previously (*2*). These persons were considered likely to have been infected with the MDRTB strain, though none demonstrated active disease. Table 1 shows that the close contact group had a TST reactor proportion of 9.09%. Table 2 shows a 3.1% TST reactor rate for the casual contact group (risk ratio [RR] 2.86, 95% confidence interval [CI] 1.22–6.74, p = 0.011). The index patient was assigned to the recruit-training platoon for 3 weeks, a rehabilitation squad for 9 days, and the separation platoon for 4 days. The TST reactor proportion for persons with >3 weeks of exposure in the recruit-training platoon was substantially lower than shorter duration of exposure in the rehabilitation and separation units (RR 0.19, 95% CI 0.05–0.66, p = 0.0032). A possible explanation for this apparent paradox would be increasing infectiousness during this later period, which is supported by progressive clinical symptoms seen in the index patient.

**Table 1:** Close contact tuberculin skin test (TST) reactor rates by exposure location

	Total	Old reactors	TST	New reactor	Reactor rate (%)
Recruit training platoon only	67	1	65	4	6.15
Recruit processing units only	53	1	48	5	10.04
Multiple exposures	38		30	4	13.33
All close contacts	158	2	143	13	9.09

**Table 2:** Tuberculin skin test (TST) reactor rate by exposure duration

	Contact duration	Total	Old reactors	TSTs placed	New reactor	Reactor rate (%)	Group reactor rate (%)
Casual contacts*	Likely none	25	0	19	1	5.26	3.2
	Possible	256	13	233	7	3.00	
Close contacts	Unknown	34	1	33	1	3.03	9.09
	>3 weeks† Sep 13–Oct 12	70	1	70	3	4.29	
	1–3 weeks Oct 12–21	42	0	31	6	19.35	
	<1 week Oct 21–26	12	0	9	3	33.33	
Total		439	15	395	21	5.32	

*The close contacts were more likely to convert than the incidental contacts. Risk ratio (RR) 2.86, 95% confidence interval (CI) 1.22–6.74, p = 0.011. †The close contacts with >3 weeks of exposure were less likely to convert than those with <3 weeks of exposure. RR 0.19, 95% CI 0.05–0.66, p = 0.0032.

The optimal treatment protocol for new TST reactors from likely MDRTB sources is undefined, which leads to extensive consultation with TB experts to determine treatment timing and medications (*3**–**10*). The imminent transfer of reactors to new duty stations and the upcoming holiday leave period complicated the recommendations. Timing options included the following: 1) start medication immediately, retain reactors on base 7–10 days to verify medication tolerance, and allow self-medication during the transfer to their next duty station; 2) start medication immediately, allow self-medication during holiday leave, and continue therapy at their next duty station; or 3) delay treatment until reactors complete 2–3 weeks of holiday leave and initiate treatment at their next duty station. Ultimately, the graduating recruits were allowed holiday leave and began therapy at their next duty station.

Because the index patient's isolate was resistant to isoniazid and rifampin, several medication options were considered. The literature was reviewed and options assessed for medications, adverse effects monitoring (clinical vs. biochemical), duration (4, 6, 9, 12, or 24 months), and self-administered versus directly observed therapy. Three options emerged: 1) no medication with close clinical and radiologic monitoring for 2–3 years; 2) monotherapy with a fluoroquinolone; or 3) two-drug regimen consisting of pyrazinamide and a fluoroquinolone. This third option, initially strongly considered from prior recommendations (*4*), was not chosen because published case series suggested poor tolerance and unacceptable hepatotoxicity (*8**,**9*). By consensus, US Navy and Centers for Disease Control and Prevention (CDC) infectious disease specialists recommended a fluoroquinolone for at least 12 months. In vitro studies suggest that gatifloxacin and moxifloxacin have greater activity against *Mycobacterium tuberculosis* than older fluoroquinolones, though treatment efficacy for latent TB infection has not been documented in the literature (*11**,**12*). Ultimately, gatifloxacin was selected based on availability on the Department of Defense formulary. Therefore, the recruit reactors at high risk for latent TB infection from the MDRTB isolate were counseled, and 400 mg gatifloxacin was administered orally daily. Although the Food and Drug Administration had not approved gatifloxacin to treat TB, this protocol represented the most appropriate therapy, based on the limited data available.

Upon arrival for training, recruits receive a single-step TST and have historically demonstrated a baseline TST reactor proportion of 0.35% (*13*). However, several of the reactors in the casual exposure category were not recruits and had vague and limited exposure histories. For example, 1 reactor drove a bus that the index patient may have ridden. Persons in these positions do not routinely undergo TST screening and would be in populations with unknown TST conversion rates. Using the "concentric ring approach," further investigation on base was deferred since the conversion proportion of personnel with positive TST results could not be separated from the background level in the local population (*2*). Military personnel would continue to receive TST surveillance consistent with the most current Navy medicine policy (*1*).

Only 6 of the 13 reactors in the higher-risk groups remained on active duty, and their transfer required explicit coordination to ensure appropriate follow-up. In collaboration with CDC, military preventive medicine personnel communicated with 5 state departments of health to ensure appropriate follow-up for the other 7 TST reactors in the high-risk group. More than 30 state health departments were notified of other casual contacts that were dropped from training.

## Conclusions

This contact investigation illustrates the complexities associated with the public health management of MDRTB exposures in military recruit training settings. It demonstrates the importance of close coordination of efforts among military medical personnel, expert tuberculosis consultants, CDC, and state health departments in such cases. It shows some of the uncertainties in the clinical management of reactors associated with exposure to MDRTB sources, exacerbated in this case by military related factors. It highlights the complexities associated with public health management of MDRTB exposure and demonstrates the necessity of response preparedness, close consultation, communication, and coordination of efforts. This outbreak preceded recently published guidance on TB investigations and treatment, although it was generally handled consistent with that guidance (*14**,**15*).
